# Annual Meeting of the Northern Ohio Dental Association

**Published:** 1890-06

**Authors:** 


### PROCEEDINGS.

### Annual Meeting of the Northern Ohio Dental Association.

The thirty-first annual meeting of the Northern Ohio Dental Association was held in Canton, Ohio, May 13th, 14th and 15th, at Council Chamber. About forty members were present at the session, and a most interesting meeting was had. The meeting was called to order by the Vice-President, F. S. Whitslar, of Youngstown, and after the preliminary exercises were had the following papers were read and discussed :

Combination Fillings; Their Value and Where Indicated, paper, by Dr. Chas. R. Butler, Cleveland. The discussion was opened by Drs. S. B. Dewey, Cleveland, and F. S. Whitslar, Youngstown, O.

Prosthetic Dentistry of To-day, paper by Dr. Geo. H. Wilson, Painesville, O. The discussion was opened by Dr. J. E. Phelps, of Chagrin Falls. The papers and discussions excited the liveliest and best work of the society, and we regret that the discussions were not taken by a stenographic reporter.

The second day was devoted to clinics consisting of a setting of a Dewey crown by H. F. Harvey ; immediate root filling, by Jno. B. Bell, and contour gold filling with electric mallet by Geo. H. Wilson. A visit to the Deuber Watch Works completed the second day's work. In the evening a valuable paper by A. J. Douds, of Canton, was read, subject: Care of Children's Teeth. The paper will appear soon in this journal. At Thursday noon the meeting closed. The following officers were elected for the next year: President, F. S. Whitslar, of Youngstown ; Vice-President, F. H. Lyder, of Akron ; Recording Secretary, F. F. Douds, of Canton ; Corresponding Secretary, R. H. Barnes, of Cleveland ; Treasurer, Chas. Buffett, of Cleveland.

The next meeting will be held at Oberlin, the second Tuesday in May, 1891. An interesting programme is made out and will prove a good meeting. The Northern Ohio Society is increasing in its work in the science of dentistry. W.

